# Prediction of Concomitant Nosocomial Infection in Patients Previously Colonized Colorectally by Multidrug-Resistant Bacteria in an SDD Setting

**DOI:** 10.3390/antibiotics13080717

**Published:** 2024-07-30

**Authors:** Sergio Ruiz-Santana, José Dearriba-Reyes, Pedro Saavedra, Laura Iglesias-Llorente, Laura Alonso-Acero, Carmen-Rosa Hernández-Socorro, Catalina Sánchez-Ramírez

**Affiliations:** 1 Intensive Care Unit, Hospital Universitario de Gran Canaria Dr. Negrín, University de Las Palmas de Gran Canaria, E-35010 Las Palmas de Gran Canaria, Spain; csanrams@gobiernodecanarias.org; 2 Department of Medical and Surgical Sciences, University de Las Palmas de Gran Canaria, E-35010 Las Palmas de Gran Canaria, Spain; jose.dearriba101@alu.ulpgc.es; 3 Department of Mathematics, University de Las Palmas de Gran Canaria, E-35010 Las Palmas de Gran Canaria, Spain; pedro.saavedra@ulpgc.es; 4 Department of Microbiology, Hospital Universitario de Gran Canaria Dr. Negrín, E-35010 Las Palmas de Gran Canaria, Spain; liglllo@gobiernodecanarias.org (L.I.-L.); laloace@gobiernodecanarias.org (L.A.-A.); 5 Department of Radiology, Hospital Universitario de Gran Canaria Dr. Negrín, University de Las Palmas de Gran Canaria, E-35010 Las Palmas de Gran Canaria, Spain; carmenrosa.hernandez@ulpgc.es

**Keywords:** colorectal colonization, infection control, decontamination, drug resistance, bacterial, pneumonia

## Abstract

Background: Antibiotic resistance is a worldwide concern. This study retrospectively analyzed patients admitted to the ICU of a tertiary hospital over a period of 7 months who were rectally colonized by multidrug-resistant microorganisms. The incidence of concomitant nosocomial infections was estimated, thus providing the risk of a colonizing microorganism producing a nosocomial infection. Methods: Infections with the same microorganism (concomitant) or different microorganisms (non-concomitant) were analyzed in order to adjust the empirical antibiotic treatment. Patients with rectal colonization by at least one multidrug-resistant bacterium (MDRB) on admission or after ICU admission were included. All patients had complete selective digestive decontamination (SDD) prophylaxis. For univariate analysis, categorical variables are expressed as frequencies and percentages and continuous variables as means and standard deviations, or as medians and interquartile ranges. For multivariate analysis, the model is summarized with *p*-values and hazard ratios with 95% confidence intervals. Survival analysis was conducted using the Kaplan–Meier method, which was performed to evaluate the time elapsed from colonization to infection by the same bacteria. Statistical significance was considered at *p* < 0.05. Results: Of the 130 patients with MDRB bacterial colonization analyzed, 98 remained free of infection, while 22 developed non-concomitant infections and 10 had infections concomitant to rectal colonizing bacteria. OXA-48-producing bacteria and MDR-*Pseudomonas* spp. incidences were 18.9% (95% CI: 7.96–35.2) and 44.4% (CI: 13.7–78.8), respectively. Conclusions: OXA-48-producing bacteria and MDR-*Pseudomonas* spp. were the only bacteria associated with the development of infections concomitant to rectal colonization in an SDD setting. The incidence of MDRB infections was low.

## 1. Introduction

Sheila Alexander was one of the first patients treated with penicillin, a revolutionary drug that offered the opportunity to relegate the bacterial diseases that threatened humanity to history. Since then, antibiotic resistance has been a concern for nearly 85 years, arising in parallel with Sheila’s treatment, although it was not until the late 1940s that a direct link between antibiotic exposure and antibiotic resistance was recognized [1].

The mechanisms of resistance by which microorganisms can become resistant to an antibiotic agent and the number of antibiotic-resistant species are increasing [2]. Mortality and unfavorable outcomes escalate whenever antibiotics are used and misused [3]. Therefore, although drug resistance is an inevitable consequence of drug use, it is important to manage it through accurate diagnosis and tailored treatment regimens [4,5]. It is our duty, as healthcare professionals, to prevent the further uncontrolled spread of antibiotic resistance [6].

The overall incidence rates of nosocomial infections in the ICU, including bacteremia secondary to infection from other foci, included in the Nosocomial Infection Surveillance Study-Hospitals in Spain (ENVIN-HELICS) were 7.85% and 10.64‰ days of stay in 2023 [7].

To mitigate the effects of antibiotic resistance, selective digestive decontamination (SDD) has been implemented in a considerable number of intensive care units (ICUs) in the Netherlands and Spain in recent years. This is a prophylactic treatment for critically ill patients based on an oropharyngeal paste and enteral suspension containing antimicrobials [8]. The patient sample studied in this article was presented to an ICU with an SDD setting. The application of SDD has led to a reduction in nosocomial infection rates. In addition, patients with multidrug-resistant bacteria (MDRB) infections decreased significantly in the ICU of our hospital, which had high rates of resistance before the use of SDD [8,9]. In addition, it has been shown that, in SARS-CoV-2-infected patients, the application of SDD in well-established infection control programs significantly reduced the incidence of ventilator-associated pneumonia and MDRB infections, along with a non-significant reduction in the incidence of secondary bacteremia [10].

Nosocomial infections in patients colonized by MDRB in the colon or rectum pose a therapeutic dilemma. There is insufficient scientific evidence to determine when broad-spectrum antibiotic therapy should be administered empirically to treat MDRB, or when a more conservative approach should be taken to avoid further antibiotic resistance [5].

The aim of this study is to evaluate the incidence of nosocomial infections caused by the same microorganism (concomitant) or by different microorganisms (non-concomitant) in patients colonized rectally by MDRB at the time of admission to the ICU or after admission to a tertiary referral hospital.

## 2. Results

Among the 130 patients with MDRB rectal colonization included in the study, 98 did not develop any MDRB infection, while 32 did. Of the latter, MDRB infections were caused by microorganisms other than those colonizing the rectum in 22 patients, whereas 10 developed an infection due to the MDRB colonizing the rectum. No patient had more than one infection with a concomitant MDRB.

Patient characteristics are shown in Table 1. Most patients were male (69.2%), with a median age of 64 years. Within the cohort of MDRB concomitant infections, the median time between identification of rectal colonizing microorganisms and diagnosis of concomitant infection was 6 days, with the interquartile range between 4.5 and 13.75 days.

There were statistically significant differences between cohorts in several variables, such as APACHE-II score (*p* < 0.001). Most of the patients received antibiotics 48 h before admission (70.8%), some of them also received parenteral nutrition (8.5%), and both patient groups were associated with MDRB infections (*p* < 0.05). Renal replacement therapy patients also showed significant association with MDRB concomitant infections (*p* < 0.001).

Patients were generally well-balanced across several comorbidities and clinical parameters, with no statistically significant differences observed between groups.

No patients were found in the following groups: tuberculosis, MRSA, MDR-*Acinetobacter* spp., VRE, ventricular shunt, and malnourished patients.

Table 2 summarizes the prevalence of rectal colonization by several bacteria at and after ICU admission. Statistically significant differences were observed among patients rectally colonized by MDR-*Pseudomonas* spp. (*p* = 0.031), OXA-48-producing Enterobacteriaceae (*p* = 0.015), and CRE (*p* = 0.041), which were linked to MDRB infections. Among these groups, MDR-*Pseudomonas* spp. and OXA-48-producing Enterobacteriaceae proved to be associated with concomitant MDR-infection, as opposed to CRE. However, metallo-β-lactamase-producing bacteria (*p* = 0.575) and the rest of MDR-GNB (*p* = 0.266) did not show statistically significant differences. No rectal colonization by MDR-*Acinetobacter* spp. or VRE was observed.

The incidence rates of concomitant MDR-*Pseudomonas* spp. and OXA-48-producing Enterobacteriaceae were 18.9% (7.96–35.2) and 44.4% (13.7–78.8), as displayed in Table 2.

**Figure 1:**
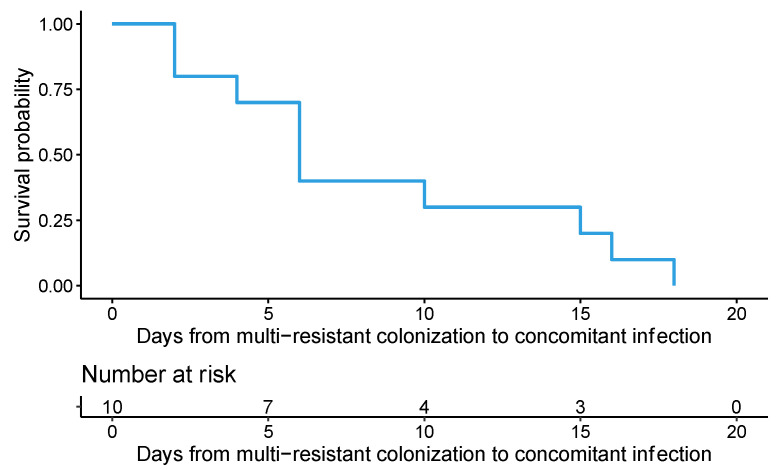
Survival function (95% CI) corresponding to the incidence of infection by MDRB concomitant with colonization. This function gives, for the entire cohort, the probability that a patient remained free of concomitant infection per day of follow-up.

The survival function (95% CI) corresponding to the incidence of infection by MDRB supposedly concomitant with colonization throughout the follow-up period is displayed in Figure 1. Initially, no patient had progressed to infection. At 10 days post-colonization, approximately 60% progressed to infection, leaving the remaining 40% free of infection.

The proportional hazard model (Cox) for the time from rectal colonization to concomitant MDRB infections is summarized in Table 3. The factors that showed independent association with this outcome were OXA-48-producing bacteria (HR = 10.1), renal replacement therapy (HR = 6.3), and rectal colonization by MDR-*Pseudomonas* spp. (HR = 8.68).

Finally, in Table 4, we display the number of concomitant MDR rectal colonizing bacteria that produced the nosocomial infections diagnosed during the study period.

## 3. Discussion

The main findings of this research are that rectal colonizations by MDR-*Pseudomonas* spp. and OXA-48-producing Enterobacteriaceae in an SDD setting were found concomitantly in MDRB infections with a significant incidence, unlike CRE, which did not show concomitance with nosocomial infection.

In recent years, there has been a significant increase in multi-antibiotic resistance, highlighting the critical importance of appropriately managing these colonizing microorganisms [11]. Furthermore, it is well known that empirical antibiotic therapy is often indispensable in reducing mortality rates in patients [11,12]. This concern prompted the present investigation into the progression of rectal colonization by MDRB and its implications for nosocomial infections.

Our findings indicate that MDR-*Pseudomonas* spp. and OXA-48-producing Enterobacteriaceae exhibit an independent higher propensity for concomitance in MDRB infections compared to other rectal colonizing MDRB. These bacteria demonstrated significant dissemination potential, even in an SSD setting. Controlling those microorganisms may reduce the risk of infection [11,12,13]. Additionally, statistical analysis revealed a notable lack of CRE rectal colonization concomitantly developing infections, indicating that these microorganisms did not lead to infection in our study patients.

Cohen et al. [14] reported an increased risk of presenting MDR-*Pseudomonas* spp. rectal colonization concomitantly with infection, with 26% of their patients colonized in the colorectal region. Furthermore, in a recent prospective study of a 1.5-year cohort of patients, Zorrilla et al. [15] showed a notable correlation between patients rectally colonized by MDR-*Pseudomonas* spp. and the onset of infection caused by this microorganism. Consistent with previous studies [14,15], the published data suggest that, when a patient has rectal colonization by an MDR-*Pseudomonas* spp., the nosocomial infection developed should be empirically treated based on the surveillance culture results antibiogram exhibited by the colonizing microorganism.

The extent of intestinal colonization by OXA-48-producing *Klebsiella pneumoniae* in hospitalized patients involves intestinal loads much higher than the *K. pneumoniae* loads published in the normal microbiota, in many cases largely replacing it [16]. Consequently—and also as expected in our cohort with OXA-48 rectal-colonizing Enterobacteriaceae—we found OXA-48-producing Enterobacteriaceae to independently and significantly produce concomitant multidrug-resistant nosocomial infections.

However, our data did not reveal an independent association between any other MDR-GNB and concomitant nosocomial infections. In our cohort, CRE infections were detected in only 1 out of 10 instances but constituted 22.7% of non-concomitant infections. This suggests that CRE can lead to multidrug-resistant infections, but these are not necessarily caused by the same colonizing rectal microorganism, at least in our investigation. Consequently, empirical broad-spectrum antibiotic therapy may not be suitable for treating infections in the context of CRE colonization.

Nevertheless, McConville et al. [17] demonstrated a different concept among adults evaluated for rectal colonization by MDRB upon admission, where the colonization status was significantly associated with the subsequent development of CRE infection. Moreover, Falcone et al. [18] observed that rectal colonization by different types of carbapenem-resistant *Klebsiella pneumoniae* led to different risks for bloodstream infections caused by the same colonizing organism. They found that patients colonized by NDM-*Klebsiella pneumoniae* were at higher risk of bacteremia compared with those colonized by KPC-*Klebsiella pneumoniae*, and that the carbapenemase type is strongly related to the specific ST clone. These investigators concluded that strategies to achieve intestinal decolonization of CRE were urgently needed. Therefore, it is conceivable that the absence of concomitance of CRE observed in our study may be attributed to the protective effect of SDD against the development of nosocomial infections [8,9,10].

The prevalence of MRSA is significantly greater in nasopharyngeal exudates compared to rectal swabs [19,20]. This could explain the absence of these microorganisms in the studied cohort.

In our series we found neither VRE nor MDR-*Acinetobacter* spp. in comparison with other authors who did find them [21,22,23]. We believe that the lower incidence of colorectal colonization in our ICU is the main reason why no cases of the aforementioned bacterial infections were documented.

In addition, our study also assessed the association between clinical characteristics and MDRB infections. Within the 48 h before ICU admission, 92 patients received antibiotic treatment. No concomitant infections were found among 95.5% of individuals in this group. Therefore, antibiotic treatment emerges as a significant risk factor for non-concomitant MDRB infection. Echoing this observation, Ceccarelli et al. [13] have emphasized the heightened susceptibility for developing nosocomial infections concomitant with colonization when antibiotics are used. A recent review has also supported this observation, recalling the importance of optimizing antibiotic treatment in such patients [5].

Moreover, the proportion of patients under renal replacement therapy was significantly higher among the concomitantly infected patients (50% of concomitant infections). Reflecting these results, Papafotiou et al. [24] obtained comparable findings in a study on risk factors for infection among a large sample of patients rectally colonized by CRE. Consequently, renal replacement therapy arises as an independent risk factor for the time to concomitant MDRB infection.

When it comes to mortality, Ceccarelli et al. [13] uncovered a significant insight in their study: they observed that the presence of CRE did not show a clear association with increased mortality, although they attributed this finding to the relatively younger age distribution within their study population. Even though our study did not yield statistically significant differences in mortality rates (*p* = 0.176), it is worth noting that a noticeable trend towards higher mortality was observed among infected patients—particularly those concomitantly presenting bacterial strains from rectal colonization. The median age was 64 years, with a higher range observed among those who developed infections (ranging from 69 to 70 years), highlighting the advanced age of the patients included in our study.

To prevent the proliferation of colonizing MDRB in the gut, SDD was employed, as it has demonstrated efficacy in preventing or eradicating the pathogenic flora carrier status in patients, which could potentially lead to nosocomial infections [23]. In line with recent evidence, we suggest that one of the factors contributing to the relatively low incidence of concomitant MDRB in infections is attributed to the protective effects conferred by SDD [19,20].

Relevant recent research such as the Australian SuDDICU study showed in secondary microbiological outcomes that there was a statistically significant decrease in the proportion of patients with positive cultures for resistant microorganisms (23.1% vs. 34.6%; absolute difference −11% [95% CI −14.7% to −7.3%]) in SDD patients vs. standard care patients [25]. Given its minimal adverse effects and the absence of association with increased antibiotic resistance in recent reviews [26], we contend that SDD represents a distinct option in the prevention of bacterial concomitance from the colon.

We also conducted a Kaplan–Meier analysis to examine the time elapsed between rectal colonization by MDRB and the subsequent development of a concomitant infection. This analysis provided valuable insights into the relationship between colonization and the dynamics of nosocomial infections. The survival function depicts the incidence of infection by MDRB concomitant with colonization. Our findings revealed that the probability of remaining free of concomitant infection decreases progressively as the follow-up duration increases, indicating an escalating risk of infection over time. Notably, all patients with nosocomial infection by MDRB concomitant with colonization acquired a nosocomial infection before reaching the twentieth day of their ICU stay.

This study presented several limitations. First, it was a retrospective observational study conducted at a single center. This design inherently limits the generalizability of the findings beyond the specific SDD setting studied. Second, the rectal colonization number, except for MDR-GBN and OXA-48-producing Enterobateriaceae, is low, which lead to large confidence intervals. Further investigations into factors influencing infection susceptibility and progression in critically ill patients are needed.

## 4. Material and Methods

### 4.1. Design and Population

A retrospective cohort study was conducted in the ICU of a tertiary reference hospital in Spain. A total of 130 patients admitted to the ICU who received SDD treatment were studied longitudinally from 1 January to 31 July 2023. We included all patients who tested positive for MDRB rectal colonization during the study period in at least one culture at admission or during ICU stay. Patients were classified as follows: 1. not infected; 2. none of the microorganisms associated with the infection were rectal colonizers (non-concomitant); or 3. at least one of the microorganisms associated with the infection was also a rectal colonizer (concomitant).

### 4.2. Study Procedures and Definitions

Colorectal surveillance samples were collected at admission and once weekly thereafter during the ICU stay. Any other additional clinical samples were obtained at the discretion of the attending physician.

ICU-acquired infections either at admission or after the ICU stay were collected from the ENVIN-HELICS registry—a national multicenter data collection system designed to record infections in ICU patients. Clinical definitions of bacterial infections are also described (http://hws.vhebron.net/envin-helics/Help/Manual_2023.pdf, accessed on 17 January 2024) [7].

Infections caused by the studied MDRB included the following [7]:

1. Enterobacteriaceae spp. resistant with extended-spectrum β-lactamase (ESBL) refers to all cases in which the patient showed colonization or infection by Enterobacterales producing extended-spectrum beta-lactamases. In general, Enterobacterales that are resistant to third-generation cephalosporins are considered to be beta-lactamase producers and would fall under this definition.
2. MDR-*Pseudomonas* spp. refers to all cases in which the patient showed colonization or infection by *Pseudomonas* spp. resistant to three or more families of antibiotics (carbapenems, cephalosporins, piperacillin-tazobactam, quinolones, or aminoglycosides) [15].
3. Carbapenem-resistant Enterobacteriaceae (CRE) refers to all cases in which the patient showed colonization or infection by Gram-negative bacilli producing carbapenemases (which confer resistance to carbapenems) before or during their stay in the ICU. Excluded from this group are OXA-48-producing Enterobacteriaceae and metallo-β-lactamase, which were separately analyzed.
4. Oxacillinase-48 (OXA-48)-producing Enterobacteriaceae.
5. Metallo-β-lactamase-producing Enterobacteriaceae.
6. Gram-negative multidrug-resistant bacteria (MDR-GNB) refer to all cases in which the patient showed colonization or infection by Gram-negative bacilli resistant to three or more families of antibiotics. This includes other Gram-negative bacilli meeting this condition and not included in previous categories.
7. Methicillin-resistant *Staphylococcus aureus* (MRSA).
8. Any strain of *Acinetobacter* spp. resistant to carbapenems.
9. *Clostridioides difficile* refers to all cases where the patient had *C. difficile* infection, determined using standard microbiological methods and requiring isolation and treatment.
10. Vancomycin-resistant *Enterococcus* spp. (VRE).

Infection was defined as those cases occurring after 48 h of ICU admission and after testing positive for colorectal colonization by MDRB. The diagnostic criteria established for the ENVIN-HELICS project were used [7]. The included infections were as follows:

1. Nosocomial pneumonia, whether associated or not with mechanical ventilation;
2. Urinary tract infection;
3. Tracheobronchitis, whether associated or not with mechanical ventilation;
4. Primary or secondary bacteremia.

Bacterial colonizations: the most relevant pathogenic species in clinical and epidemiological terms have been investigated in rectal swabs: vacomycin-resistant *Enterococcus* spp. (VRE); Enterobacteriaceae producing extended-spectrum beta-lactamases (ESBLs) or plasmid-mediated AmpC beta-lactamases (pAmpC); carbapenemase-producing Enterobacteriaceae (CPE); multidrug-resistant A. baumannii; and multidrug-resistant *Pseudomonas* spp. (MDR-*Pseudomonas* spp.) [27].

#### 4.2.1. SDD Protocol

A well-established full SDD protocol commonly used in clinical practice in Spain was applied [9]. SDD was initiated in patients that were mechanically ventilated for more than 48 h and was administered until the patients were discharged from the ICU. This regimen consisted of three components: (1) 1 g of an oral paste applied in the oral cavity, composed of 20 mg of 2% colistin, 30 mg of 3% tobramycin, and 20 mg of 2% nystatin; (2) 14 mL of a suspension containing 140 mg of 1% colistin, 180 mg of 2% tobramycin, and 453.6 mg of 3.2% nystatin, which was administered into the intestine; and (3) cefotaxime (or levofloxacin in the case of allergy), which was administered during the first 4 days of treatment with SDD. In patients with methicillin-resistant *Staphylococcus aureus* (MRSA), a solution consisting of 40 mg of 4% oropharyngeal paste and 700 mg of vancomycin in a digestive solution was added to the above-mentioned regimen. We applied paste and enteral solution every 8 h until discharge.

#### 4.2.2. Other Definitions to Consider

Immunosuppressed patients were characterized as individuals diagnosed with either primary or acquired immunodeficiency disorders or suffering from conditions sufficiently advanced to compromise their defenses against infections.

Neutropenic patients were identified as those with absolute neutrophil counts below 500 at any point during their ICU admission.

The variable “neoplasm” encompassed patients diagnosed with solid organ or hematological malignancies within the past 5 years.

Malnourished patients were defined as individuals with serum albumin levels below 30 g/L upon admission.

For patients with renal failure, cirrhosis, chronic obstructive pulmonary disease (COPD), diabetes mellitus, or COVID-19, their medical history was reviewed and considered as such.

The information was extracted from the ICU Service database using the hospital Critical Care Manager (Harris Computer, Peabody, MA, USA, version number 8.6) and DRAGO^®^ software (CGM Clinical España S.L.U., Madrid, Spain, version number SP17.02A16).

### 4.3. Variables Collected

On admission to the ICU, we recorded demographic, clinical, and analytical data; scores to assess severity of illness in critically ill patients using Acute Physiology and Chronic Health Disease Classification System II (APACHE-II) [26]; and comorbidity scores using the Charlson Index [27]. Nosocomial infection-dependent data were also collected, including microorganisms, type of infection, use of SDD, and date of diagnosis of infection.

### 4.4. Microbiological Methods

Detection of MDRB colonization in patients was performed through culturing of rectal swabs using differential and/or selective media: chromID VRE (bioMérieux, Marcy-l’Etoile, France), chromID CARBA SMART (bioMérieux, France), and MacConkey agar supplemented with 4 mg/liter cefotaxime [28,29,30]. The investigation of MDRB was performed at the time of admission and weekly during patients’ stay in the ICU.

The isolates were identified to the species level first using the MALDI Biotyper Compass 4.1.14 software BDAL version 10 (Bruker Daltonik, Bremen, Germany) following the manufacturer’s instructions.

Antimicrobial susceptibility testing (AST) in surveillance samples was performed by disk diffusion on Muller–Hinton agar plates with a standard inoculum of the test organism that corresponds to 0.5 McFarland turbidity as described by EUCAST (European Committee on Antimicrobial Susceptibility Testing) and CLSI (Clinical and Laboratory Standards Institute) [31,32]. AST in clinical samples was determined by Vitek^®^2 (BioMérieux, Marcy l’Etoile, France) cards and antibiotic gradient strips by Etest^®^ (BioMérieux, Marcy l’Etoile, France) or Liofilchem MIC Test Strips (Liofilchem, Roseto degli Abruzzi, Italy) in accordance with the manufacturers’ recommendations. We used EUCAST guidelines for detection of resistance mechanisms and specific resistances of clinical and/or epidemiological importance [33]. We confirmed the type of carbapenemases by polymerase chain reaction: Xpert^®^ Carba-R (Cepheid, Inc., Sunnyvale, CA, USA) or MDR Direct Flow Chip Kit (Vitro, Sevilla, Spain).

### 4.5. Statistical Analysis

#### 4.5.1. Univariate Statistical Analysis

Categorical variables are expressed as frequencies and percentages and continuous variables as means and standard deviations (SDs) when the data followed a normal distribution, or as medians and interquartile ranges (IQR = 25th–75th percentile) when their distribution departed from normality. The percentages were compared, as appropriate, using the Chi-square ($\chi^{2}$) test or the exact Fisher test, the means using the *t*-test, and the medians using the Wilcoxon test for independent data. Multiple comparisons were carried out according to (a) Conover’s All-Pairs Rank Comparison Test for medians [34] or (b) G-test rates.

#### 4.5.2. Time to the Concomitant Infection

To evaluate the time elapsed from the identification of colonization by a multi-resistant microorganism to infection by the same microorganism (concomitant infection), a survival analysis was performed. First, when a patient did not develop a multi-resistant infection concomitant to colonization, the time elapsed between colonization and the end of follow-up was considered as a right-censored observation. The survival function for the times to concomitant infections was estimated by means of the Kaplan–Meier method. Identification of factors associated with time to concomitant infection was performed using a proportional hazards model. The model is summarized using *p*-values and hazard ratios (HR), which were estimated using 95% confidence intervals.

Statistical significance was set at *p* ≤ 0.05. Data were analyzed using the R package, version 4.2.1 (R Development Core Team, 2022) [35]. The incidences of nosocomial infections associated with concomitant microrganisms were estimated by 95% confidence intervals using an exact method.

## 5. Conclusions

Rectal colonization by MDR-*Pseudomonas* spp. and OXA-48-producing bacteria were the only bacteria associated with the development of concomitant rectal colonization by nosocomial MDRB infections in an SDD setting. There was also a lack of correlation between CRE colonization and concomitant microorganisms in nosocomial infections. Finally, the incidence of MDR infections in the study cohort was low. These facts have to be considered when guiding empirical antibiotic treatment in cases of concomitant rectal colonization by MDRB nosocomial infection.

## Figures and Tables

**Table 1:** Patient characteristics: overall and by multidrug-resistant infection group.

		Multidrug-Resistant Infections			
	Overall *N* = 130	No *N* = 98	Non-Concomitant *N* = 22	Concomitant *N* = 10	*p*-Value
Age (years)	64 (53; 73)	62 (53; 73)	69 (56; 75)	70 (52; 74)	0.484
Sex male	90 (69.2)	70 (71.4)	13 (59.1)	7 (70.0)	0.505
APACHE-II score	16 (11; 20)	14 (9.2; 19) ^a^	21 (18; 25) ^b^	22 (16; 26.8) ^b^	<0.001
Charlson index score	3 (2; 5.8)	3 (2; 5)	4 (2.2; 6)	3.5(2.2; 4.8)	0.601
Glasgow coma score	12 (3; 15)	12 (3; 15)	10 (4.5; 15)	13.5(10; 15)	0.783
ICU death	12 (9.2)	7 (7.1)	3 (13.6)	2 (20.0)	0.176
Urgent surgery	18 (13.8)	13 (13.3)	2 (9.1)	3 (30.0)	0.259
Immunosuppressed patients	9 (6.9)	7 (7.1)	2 (9.1)	0	0.843
Neutropenic patients	3 (2.3)	2 (2.0)	1 (4.5)	0	0.575
Parenteral nutrition	11 (8.5)	3 (3.1) ^a^	5 (22.7) ^b^	3 (30.0) ^b^	<0.001
Traumatic patients	6 (4.6)	6 (6.1)	0	0	0.749
C.A.D. patients	25 (19.2)	23 (23.5)	1 (4.5)	1 (10.0)	0.085
ATB 48 h	92 (70.8)	64 (65.3) ^a^	21 (95.5) ^b^	7 (70.0) ^a^	0.009
Surgery (last 30 days)	5 (3.9)	3 (3.1)	2 (9.1)	0	0.359
*Clostridioides difficile*	2 (1.5)	1 (1.0)	1 (4.5)	0	0.433
Renal replacement therapy	11 (8.5)	3 (3.1) ^a^	3 (13.6) ^a,b^	5 (50.0) ^b^	<0.001
Renal failure	18 (13.8)	12 (12.2)	5 (22.7)	1 (10.0)	0.407
COPD	13 (10.0)	10 (10.2)	3 (13.6)	0	0.591
Cirrhosis	3 (2.3)	3 (3.1)	0	0	1
Neoplasm	5 (3.9)	4 (4.1)	1 (4.5)	0	1
Diabetes mellitus	40 (30.8)	30 (30.6)	7 (31.8)	3 (30.0)	1
COVID-19 patients	7 (5.4)	5 (5.1)	2 (9.1)	0	0.781
Impella^®^ device	1 (0.8)	0	0	1 (10.0)	0.077
ECMO	9 (7.0)	6 (6.1)	1 (4.8)	2 (20.0)	0.225
Ventricular assistance	1 (0.8)	1 (1.0)	0	0	1
Aortic counter pulse balloon	7 (5.4)	5 (5.1)	1 (4.8)	1 (10.0)	0.779
Transplant types					0.81
No	122 (93.8)	91 (92.9)	21 (95.5)	10 (100.0)	
Liver	1 (0.8)	1 (1.0)	0	0	
Heart	4 (3.1)	4 (4.1)	0	0	
HSC	3 (2.3)	2 (2.0)	1 (4.5)	0	

Data are medians (IQR) and frequencies (%). ^(a,b)^ Different superscripts show significant differences for *p* < 0.05. Abbreviations: APACHE, Acute Physiology and Chronic Health Evaluation; ICU, intensive care unit; C.A.D., coronary artery disease; ATB 48 h, antibiotics 48 h before ICU admission; COPD, chronic obstructive pulmonary disease; ECMO, extracorporeal oxygenation membrane; HSC, hematopoietic stem cells.

**Table 2:** Predictive values of concomitant nosocomial infection in patients with rectal colonization by MDRB acquired on/after ICU admission.

	Colonized	Concomitant Infection	Incidence (95% CI) *
MDR-GNB	84	0	-
CRE	12	0	-
OXA-48	37	7	18.9 (7.96–35.2)
MDR-*Pseudomonas* spp.	9	3	44.4 (13.7–78.8)
Metallo-β-lactamase	3	0	-
ESBL	6	0	-

* Incidences are %. MDR-GNB, multidrug-resistant Gram-negative bacteria; CRE, carbapenem-resistant Enterobacteriaceae; OXA-48, oxacillinase-48-producing bacteria; ESBL, extended-spectrum β-lactamase.

**Table 3:** Proportional hazard model (Cox) for the time to concomitant MDRB infection.

Factor *	*p*-Value	Hazard Ratio (95% CI)
OXA-48	0.004	10.1 (2.084; 49.2)
Renal replacement therapy	0.005	6.3 (1.77; 22.5)
Rectal colonization by MDR-*Pseudomonas* spp.	0.007	8.68 (1.8; 41.8)

(*) Factors were observed on admission. OXA-48, oxacillinase-48-producing bacteria.

**Table 4:** Classification of MDRB infections according to concomitant microorganisms.

	NP *N* = 4	UTI *N* = 1	TCB *N* = 4	BAC *N* = 1
MDR-GNB	-	-	-	-
CRE	-	-	-	-
OXA-48	2 (50)	1 (100)	3 (75)	1 (100)
MDR-*Pseudomonas* spp.	2 (50)	-	1 (25)	-

Data are frequencies (%). NP, nosocomial pneumonia; UTI, urinary tract infection; TCB, tracheobronchitis; BAC, bacteremia; MDR-GNB, multidrug-resistant Gram-negative bacteria; CRE, carbapenem-resistant Enterobacteriaceae; OXA-48, oxacillinase-48-producing bacteria.

## Data Availability

Please contact the authors for data requests.
